# Comparation of drug-eluting stents and control therapy for the treatment of infrapopliteal artery disease: a Bayesian analysis

**DOI:** 10.1097/JS9.0000000000000736

**Published:** 2023-09-14

**Authors:** Yang Li, XuWei Shen, Hui Zhuang

**Affiliations:** Xiamen Cardiovascular Hospital, Xiamen University, Xiamen City, Fujian Province, China

**Keywords:** bayesian analysis, clinical patency, drug-eluting stent, infrapopliteal arterial disease, meta-analysis, sirolimus-eluting stent

## Abstract

**Background:**

Critical limb-threatening ischaemia is a life-threatening disease which often combines with infrapopliteal arterial disease. Percutaneous transluminal angioplasty (PTA) is recommended as the first-line treatment for infrapopliteal arterial disease. Drug-eluting stent (DES) is another widely used option; however, its long-term therapeutic effect is controversial. The effectiveness of different DES for infrapopliteal arterial disease needs further exploration.

**Methods and results:**

The PubMed, EMBASE, Cochrane Library and Clinical trials were systematically searched from inception to 1 February 2023. Literatures were included if the study was original, peer-reviewed, published in English or Chinese, and contained patients diagnosed with simple infrapopliteal arterial disease or with properly treated combined inflow tract lesions before or during the study procedure. A total of 953 patients, 504 in the DES group and 449 in the PTA/bare-metal stenting (BMS) group, from 12 randomised controlled trials were included in the meta-analysis. The results showed that DES is superior to control group for improving clinical patency, reducing the restenosis rate, and reducing the amputation rate at 6 months, 1 year, and 3 years post-treatment [at 3 years, risk ratio (RR): 1.90, 95% CI 1.23–2.93; RR: 0.87, 95% CI 0.79–0.96; RR: 0.60, 95% CI 0.36–1.00, *P*=0.049]. In addition, subgroup analyses suggested that DES is superior to BMS and PTA in improving clinical patency and reducing target lesion revascularisation and restenosis rates at 6-month and 1-year post-treatment. The network meta-analysis indicated that sirolimus-eluting stent was superior for improving clinical patency (at 1 year, RR: 0.23, 95% CI 0.08–0.60) and reducing the restenosis rate (at 6 months, RR: 31.58, 95% CI 4.41–307.53, at 1 year, RR: 3.80, 95% CI 1.84–8.87) significantly. However, according to the cumulative rank probabilities test, everolimus-eluting stent may have the lowest target lesion revascularisation rates and amputation rates at 1-year post-treatment (the cumulative rank probability was 77% and 49%, respectively).

**Conclusions:**

This systematic review and network meta-analysis showed that DES was associated with more clinical efficacy than PTA/BMS significantly. In addition, sirolimus-eluting stent and everolimus-eluting stent may have better clinical benefits.

## Introduction

Highlights

- The first-line treatment for infrapopliteal arterial disease is percutaneous transluminal angioplasty (PTA).
- Percutaneous transluminal angioplasty/bare-metal stenting is not as clinically effective as the use of drug-eluting stents for long-term follow-up.
- Different anti-proliferative drugs in drug-eluting stent may result in different clinical outcomes.

Critical limb-threatening ischaemia (CLTI) is a life-threatening disease characterised by chronic pain at rest and tissue loss. Approximately 30% of patients with CLTI require major amputation, while limb preservation may be possible within 1 year of diagnosis in 50% of patients. However, up to 50% of patients die within 5 years of diagnosis^1^. Patients with CLTI often present with infrapopliteal arterial disease. Due to its anatomical characteristics, the treatment of infrapopliteal arterial disease is challenging^2^.

Improvements in endovascular intervention technology have allowed for angioplasty and stent implant to be used as first-line therapies for patients with CLTI^1^. Common clinical treatments for infrapopliteal arterial disease include percutaneous transluminal angioplasty (PTA), drug-coated balloon (DCB), bare-metal stents (BMS), and drug-eluting stents (DES)^3^. PTA is the first-line treatment for infrapopliteal arterial disease^4^. DCB is widely used clinically due to its inhibitory effects on intimal proliferation. However, restenosis may still occur in the infrapopliteal arteries after PTA or DCB placement due to the lack of necessary vascular support^5,6^. BMS and DES can provide adequate vascular support. DES can effectively overcome elastic recoil and flow-limiting dissection and reduce neointimal hyperplasia-derived restenosis with the anti-proliferative drug coating.

Several studies and meta-analyses have evaluated the efficacy of DES for the treatment of infrapopliteal arterial disease^7–10^. However, some meta-analyses included patients with arterial lesions at locations other than the infrapopliteal arteries, and few studies regarding the treatment differences between DES and PTA, BMS, or DCB have been reported. In addition, coronary DES are typically used to treat infrapopliteal arterial disease, and the types of anti-proliferative drug coating and stent structures are complex. Therefore, the therapeutic effects of different DES require investigation.

This study aimed to perform a meta-analysis of randomised controlled trials (RCT), comparing the effects of DES to those of other endovascular techniques, and a network meta-analysis to investigate the effectiveness of different DES treatment options for infrapopliteal arterial disease.

## Materials and methods

This systematic review and Bayesian analysis was performed according to the Preferred Reporting Items for Systematic Review and Meta-Analyses (PRISMA) (SDC 1, Supplemental Digital Content 1, http://links.lww.com/JS9/A976, Supplemental Digital Content 12, http://links.lww.com/JS9/A987) and registered with the international prospective register of systematic reviews^11^. This work has also been reported in line with Assessing the Methodological Quality of Systematic Reviews 2 Guidelines (AMSTAR-2, Supplemental Digital Content 2, http://links.lww.com/JS9/A977)^12^.

### Search strategy and selection criteria

We obtained eligible clinical studies by searching the PubMed, EMBASE, Cochrane Library and Clinical trials from their respective dates of inception to 1 February 2023. The core search terms included ‘peripheral arterial disease’, ‘critical limb-threatening ischemia’, ‘infrapopliteal arterial disease’, ‘below the knee’, ‘stent’, and ‘drug eluting stent’. Based on research quality considerations, the study type was set to RCT. We adjusted the search for each database as needed. The search strategy performed for PubMed is detailed in SDC 2, Supplemental Digital Content 3, http://links.lww.com/JS9/A978. This review included literatures that are original, peer-reviewed, published in English or Chinese, and reported clinical papers. Patients in eligible studies should be diagnosed simple infrapopliteal arterial disease. Studies containing patients with combined inflow tract lesions, such as superficial femoral and iliac artery lesions, were also included if inflow tract lesions were treated properly without complications, either before or during the procedure of study. Studies were excluded if they were not clinical studies (such as letters, case reports, or case series) or single-arm studies. The efficacy outcomes of interest included: (1) clinical patency; (2) restenosis rate (vascular stenosis ≥50%); (3) target lesion revascularisations (TLR); (4) ankle brachial index (ABI); (5) all-cause death; and (6) target limb amputations (minor limb amputations: foot, toes; major limb amputations: above ankle). Relevant systematic review and meta-analyses were screened during the search process to make sure additional eligible studies were included.

### Data extraction and quality assessment

Eligible studies retrieved through the search strategy were screened by two investigators (A and B). After evaluating the titles and abstracts, investigators assess remaining eligible studies with full text reading. A third investigator (C) resolved any disagreements during the review process. Following the Cochrane Handbook’s recommendations, investigators A and B extracted the necessary information from the included studies and assessed the methodological quality of eligible studies. Investigator C evaluated eligible randomised controlled trials using the Cochrane tool.

### Data synthesis and analysis

Investigators A and B collected original four-table data or continuous variable relate data, while a third investigator (C) verified the data for accuracy. We used Stata 12.0 software to combine and calculate the overall effect size, 95% CI, *P* value, and the weight of each study. The statistical heterogeneity was assessed by Q tests and I^2^ statistics. If *P* value less than 0.1 or I^2^ greater than 50%, which means great heterogeneity between studies, a random-effects model (Der Simonian–Laird method) was used. Subgroup analysis, or meta-regression analysis if needed, were used to analyse heterogeneity. If *P* value greater than 0.1 or I^2^ less than 50%, a fixed-effects model (Mantel–Haenszel method) was used. Publication bias was evaluated by the Harbord’s modified test for binary variables or the Egger’s modified test for continuous variables. A publication bias evaluation was performed when the number of enroled articles was greater than 2; *P* values greater than or equal to 0.1 indicated no publication bias. Sensitivity was evaluated using a meta-trim operation for binary variables and a meta-inf operation for continuous variables.

We performed a network meta-analysis within Bayesian framework. A fixed-effects model was used after we made sure it resulted in a more parsimonious result than a random-effects model. All analyses were performed using four Markov chains with 100 000 iterations after a burn‐in of 20 000 and a thinning interval of 10. The Brooks–Gelman–Rubin method was used to assess the convergence. Noninformative priors were used, and consistency and inconsistency tests were performed separately. We assessed the relative probability of an intervention being among the best treatment strategies by calculating treatment rank probability. All statistical analyses were performed using the ADDIS software.

## Results

### Database search results and quality assessment

**Figure 1:**
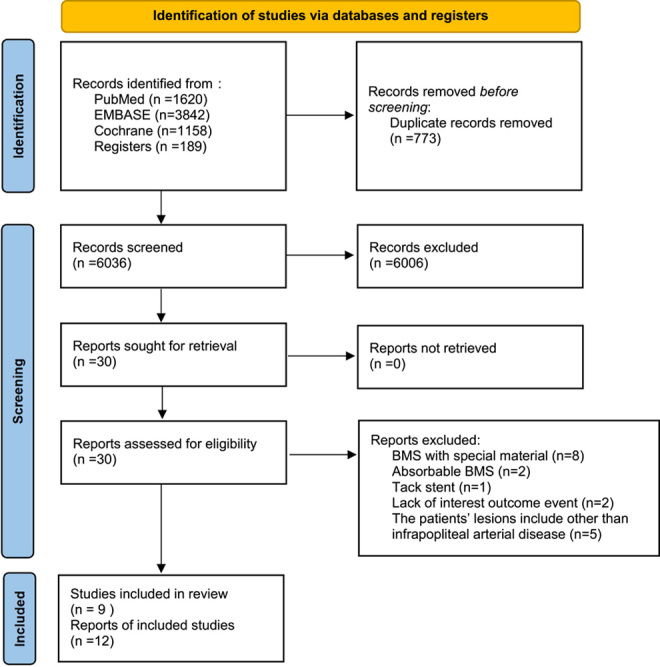
Preferred Reporting Items for Systematic Review and Meta-Analyses (PRISMA) flow diagram with reasons for study selection. BMS, bare-metal stenting.

The complete screening process and reasons for exclusion are shown in the PRISMA flow diagram (Fig. 1). Twelve RCTs were included in this systematic review and meta-analysis, including 11 that were published in English and one that was published in Chinese^13–24^. The anti-proliferative drug coatings included in these studies were sirolimus (*n*=7)^13,15,17–20,24^, everolimus (*n*=1)^14^, and paclitaxel (*n*=3)^16,22,23^. One study did not specify the type of anti-proliferative drug coating^21^, while another study was only used for systematic description of outcome events^16^.

Finally, 953 patients, 504 in the DES group and 449 in the PTA/BMS group, were included in the meta-analysis. The severity of infrapopliteal arterial disease was similar between the studies. In addition to meeting the inclusion criteria of this meta-analysis, all enroled patients had a Rutherford scale of 3–6. The target lesion length, mean reference vessel diameter, and lumen diameter had no significant differences between the experimental and control groups. The methodological quality assessment using the Cochrane Tool is shown in SDC 3, Supplemental Digital Content 4, http://links.lww.com/JS9/A979. We summarised the main characteristics of the 12 RCTs pooled in the study (SDC 4, Supplemental Digital Content 5, http://links.lww.com/JS9/A980). Enroled studies did not provide relevant results about patients’ wound, ischaemia, foot infection (WIfI) system and Global Limb Anatomic Staging System (GLASS). The baseline characteristics including proportion of males, smoking rates, history of cardiac disease, history of diabetes, target lesion length, mean reference vessel diameter, and lumen diameter are presented in SDC 4, Supplemental Digital Content 5, http://links.lww.com/JS9/A980. *P* values as described in each article indicates that the experimental and control groups had similar baseline characteristics. In 1 enroled literature, the patient population suffered from more severe vascular lesions below the knee, which were graded as Rutherford 5–6^24^. Patients younger than 18 years of age and patients who were pregnant were excluded in all literatures. Inclusion and exclusion criteria of enroled literatures were summarised in SDC 5, Supplemental Digital Content 6, http://links.lww.com/JS9/A981. Post-procedure dual antiplatelet therapy was used in all 12 RCTs, and anticoagulant therapy was only used in one RCT which was performed by heparin for 5 days after the procedure^13^. The anticoagulant and antiaggregant use and the information of stents in separated studies are summarised in SDC 6, Supplemental Digital Content 7, http://links.lww.com/JS9/A982. The network of the included studies was not well connected due to the limitations of the research, and consistency and inconsistency tests were performed separately. To evaluate the therapeutic differences of DES and PTA/BMS (control group), the DES were classified as sirolimus-eluting stent (SES), everolimus-eluting stent (EES), or paclitaxel-eluting stent (PES), and the control groups were combined in the PTA/BMS group.

### Outcomes

The results of the meta-analysis are provided in Table 1. Subgroup analyses were performed according to the treatment methods used in the control groups. Due to the insufficiency of studies, few subgroups were not available for meta-analysis, and we presented results from a single study in Table 1 separately. The publication bias evaluation was summarised in the following section and recorded the *P* value in Table 1. A sensitivity analysis was also performed and summarised in the following section.

**Table 1:** The results of meta-analyses and subgroup analyses for interest outcomes.

Outcomes	Following		RR (95% CI)/SMD (95% CI)	*P* value	Meta-analysis model	I^2^, publication bias in overall sections
Clinical patency	6 months	DES vs. BMS	1.27 (1.14, 1.41)	<0.001	Fixed	55.9%, non
		DES vs. PTA±BMS	1.37 (0.95, 1.97)	0.095	Fixed	–
		Overall	1.29 (1.15, 1.45)	<0.001	Fixed	44.7%, *P*=0.191
	1 year	DES vs. BMS	1.74 (1.29, 2.36)	<0.001	Random	72.9%, non
		DES vs. PTA	1.34 (1.13, 1.60)	0.001	Fixed	0.0%, non
		Overall	1.56 (1.28, 1.89)	<0.001	Random	64.0%, *P*=0.806
	3 years	Overall	1.90 (1.23, 2.93)	0.004	Fixed	0.0%, non
Restenosis rate	6 months	DES vs. BMS	0.18 (0.08, 0.42)	<0.001	Fixed	0.0%, non
		DES vs. PTA	0.09 (0.01, 0.63)	0.015	Fixed	—
		DES vs. P-PTA	0.48 (0.23, 1.01)	0.053	Fixed	—
		DES vs. PTA±BMS	0.51 (0.29, 0.91)	0.023	Fixed	—
		Overall	0.34 (0.23, 0.50)	<0.001	Fixed	42.8%, *P*=0.068
	1 year	DES vs. BMS	0.49 (0.39, 0.60)	<0.001	Fixed	0.0%, non
		DES vs. PTA	0.44 (0.27, 0.71)	0.001	Fixed	50.3%, non
		Overall	0.48 (0.39, 0.58)	<0.001	Fixed	0.0%, *P*=0.747
	3 years	Overall	0.87 (0.79, 0.96)	0.004	Fixed	0.0%, non
TLR	6 months	DES vs. BMS	0.24 (0.11, 0.53)	<0.001	Fixed	0.0%, non
		DES vs. PTA	0.64 (0.05, 9.03)	0.743	Fixed	—
		DES vs. P-PTA	0.56 (0.10, 3.08)	0.508	Fixed	—
		Overall	0.29 (0.15, 0.57)	<0.001	Fixed	0.0%, *P*=0.257
	1 year	DES vs. BMS	0.35 (0.19, 0.63)	<0.001	Fixed	32.2%, non
		DES vs. PTA	0.42 (0.21, 0.83)	0.013	Fixed	52.7%, non
		Overall	0.38 (0.24, 0.59)	<0.001	Fixed	21.7%, *P*=0.963
	3 year	Overall	0.54 (0.27, 1.10)	0.09	Fixed	0.0%, non
ABI	6 months	Overall	0.04 (-0.21, 0.28)	0.772	Fixed	0.0%, *P*=0.979
	1 year	Overall	0.07 (-0.14, 0.27)	0.532	Fixed	0.0%, *P*=0.207
All-cause death	6 months	Overall	1.06 (0.50, 2.25)	0.869	Fixed	0.0%, non
	1 year	DES vs. BMS	1.05 (0.65, 1.69)	0.842	Fixed	0.0%, non
		DES vs. PTA	0.85 (0.39, 1.88)	0.688	Fixed	—
		DES vs. PTA±BMS	0.93 (0.51, 1.69)	0.815	Fixed	—
		Overall	0.97 (0.69, 1.36)	0.871	Fixed	0.0%, *P*=0.187
	3 years	DES vs. BMS	0.94 (0.62, 1.41)	0.764	Fixed	0.0%, non
		DES vs. PTA	0.64 (0.05, 9.03)	0.743	Fixed	—
		DES vs. PTA±BMS	0.84 (0.52, 1.35)	0.471	Fixed	—
		Overall	0.89 (0.66, 1.22)	0.474	Fixed	0.0%, *P*=0.314
Amputation	6 months	Overall	0.57 (0.35, 0.92)	0.022	Fixed	18.3%, non
	1 year	DES vs. BMS	0.49 (0.12, 1.90)	0.299	Fixed	0.0%, non
		DES vs. PTA	0.69 (0.34, 1.38)	0.290	Fixed	—
		DES vs. PTA±BMS	0.63 (0.41, 0.98)	0.039	Fixed	—
		Overall	0.63 (0.44, 0.91)	0.013	Fixed	0.0%, *P*=0.845
	3 years	DES vs. BMS	0.59 (0.25, 1.36)	0.217	Fixed	59.5%, non
		DES vs. PTA	0.64 (0.16, 2.51)	0.525	Fixed	—
		DES vs. PTA±BMS	0.59 (0.29, 1.23)	0.162	Fixed	—
		Overall	0.60 (0.36, 1.00)	0.049	Fixed	19.1%, *P*=0.434

ABI, ankle brachial index; BMS, bare-metal stents; DES, drug-eluting stents; PTA, percutaneous transluminal angioplasty; SMD, standardized mean difference; TLR, target lesion revascularisation.

### Clinical patency

**Figure 2:**
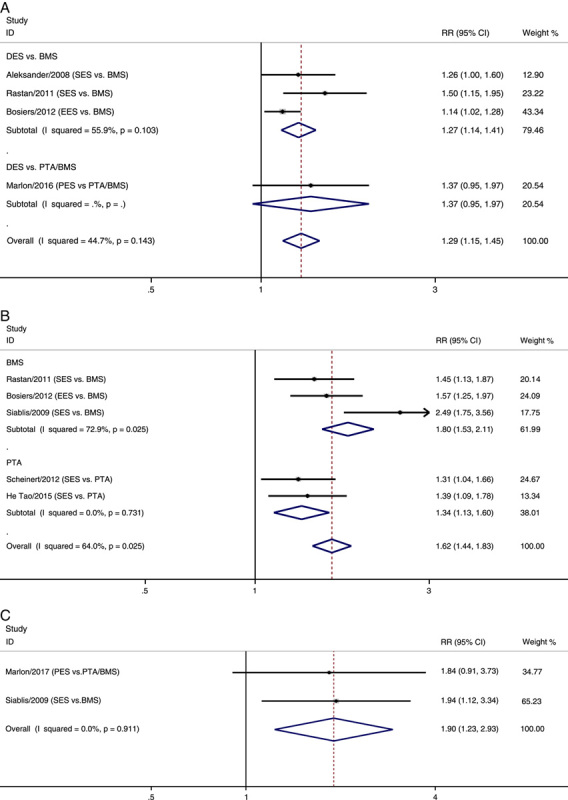
Forest plots of the estimated overall and subgroup analyses in clinical patency at 6 months (A), 1 year (B) and 3 years (C). BMS, bare-metal stenting; DES, drug-eluting stent; EES, everolimus-eluting stent; PES, paclitaxel-eluting stent; PTA, percutaneous transluminal angioplasty; RR, risk ratio; SES, sirolimus-eluting stent.

Eight RCTs reported clinical patency results, and subgroup analyses were performed according to the options of control group in further (Fig. 2)^13–15,18–20,22,23^. The use of DES had a greater effect on clinical patency than the use of PTA/BMS at 6 months, 1 year, and 3 years after treatment [risk ratio (RR) 6 months: 1.29, 95% CI 1.15–1.45; 1 year: 1.56, 95% CI 1.28–1.89; 3 years: 1.90, 95% CI 1.23–2.93]. Statistical heterogeneity among the 1-year clinical patency results was observed (I^2^=64.0%, *P*=0.025). After excluding one study^20^, the adjusted RR for primary patency at 1 years was 1.43 (95% CI: 1.27–1.62) between DES and PTA/BMS, and the heterogeneity was significantly reduced (I^2^=0.0% and *P*=0.752). We further reviewed this literature^20^ and ensured that there were no data extraction errors. Based on the results of the Cochrane Tool for assessing the risk of bias (SDC 3, Supplemental Digital Content 4, http://links.lww.com/JS9/A979), we noticed that this study^20^ showed unclear random sequence generation, allocation concealment, blinding method, and selective reporting risk which indicated that this study contained inherent flaws. DES also had a greater effect on clinical patency than BMS at 6 months and 1 year after treatment (RR 6 months: 1.27, 95% CI 1.14–1.41; 1 year: 1.74, 95% CI 1.29–2.36) and PTA after 1 year of treatment (RR 1 year: 1.34, 95% CI 1.13–1.60).

### Restenosis rate

**Figure 3:**
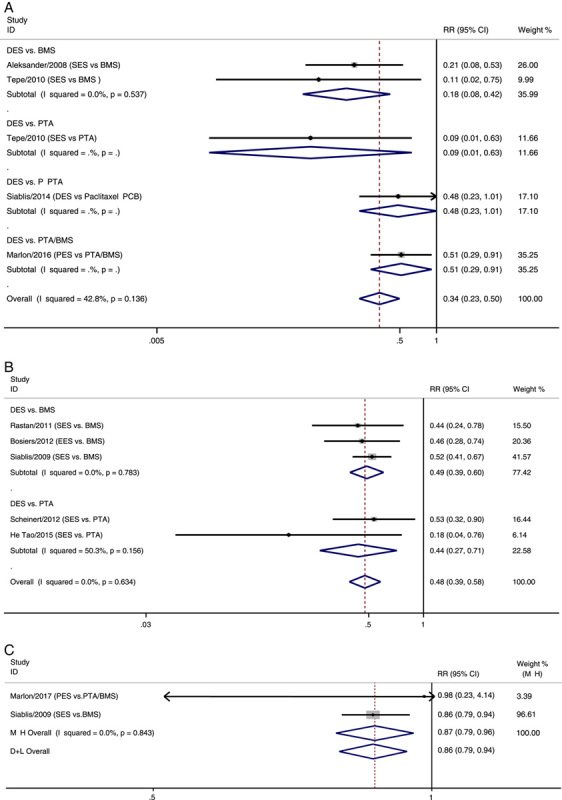
Forest plots of the estimated overall and subgroup analyses in restenosis rate at 6 months (A), 1 year (B) and 3 years (C). BMS, bare-metal stenting; DES, drug-eluting stent; EES, everolimus-eluting stent; PES, paclitaxel-eluting stent; PTA, percutaneous transluminal angioplasty; RR, risk ratio; SES, sirolimus-eluting stent.

Ten RCTs reported restenosis rates (Fig. 3)^13–15,18–24^. DES treatment significantly reduced the restenosis rate compared with PTA/BMS at 6 months, 1 year, and 3 years after treatment (RR 6 months: 0.34, 95% CI 0.23–0.50; 1 year: 0.48, 95% CI 0.39–0.58; 3 years: 0.87, 95% CI 0.79–0.96). No statistical heterogeneity was observed among the results. Subgroup analyses demonstrated that DES lowered the restenosis rate more than BMS at 6 months and 1 year after treatment (RR 6 months: 0.18, 95% CI 0.08–0.42; 1 year: 0.49, 95% CI 0.39–0.60) and PTA after 1 year of treatment (RR 1 year: 0.44, 95% CI 0.27–0.71). No statistical heterogeneity was observed in the subgroup results.

### Target lesion revascularisations

Nine RCTs reported TLR outcomes (SDC Fig. 1, Supplemental Digital Content 8, http://links.lww.com/JS9/A983)^13–15,17–19,21,23,24^. DES treatment significantly reduced the rate of TLR compared with PTA/BMS at 6 months and 1 year after treatment (RR 6 months: 0.29, 95% CI 0.15–0.57; 1 year: 0.38, 95% CI 0.24–0.59); however, there was no significant difference at 3 years post-treatment (RR 0.54, 95% CI 0.27–1.10). No statistical heterogeneity was observed among the results. Subgroup analyses demonstrated that DES treatment was associated with a significantly lower risk of TLR than BMS treatment at 6 months and 1 year after treatment (RR 6 months: 0.24, 95% CI 0.11–0.53; 1 year: 0.35, 95% CI 0.19–0.63) and PTA treatment at 1 year after treatment (RR 1 year: 0.42, 95% CI 0.21–0.83). No statistical heterogeneity was observed in the subgroup results.

### Ankle brachial index

Four RCTs compared the ABI for DES and PTA/BMS at 6 months and 1 year after treatment (SDC Fig. 2, Supplemental Digital Content 9, http://links.lww.com/JS9/A984)^13,15,18,22^. However, there were no significant differences between the ABI of the DES and control groups (standardized mean difference 6 months: 0.04, 95% CI: −0.21, 0.28; standardized mean difference 1 year: 0.07, 95% CI: −0.14, 0.27). Due to the limited number of studies, subgroup analyses could not be performed. No statistical heterogeneity was observed among the results.

### All-cause mortality

Ten RCTs reported all-cause mortality outcomes (SDC Fig. 3, Supplemental Digital Content 10, http://links.lww.com/JS9/A985)^14,16–24^. Nine of them were included in the meta-analyses^14,17–24^. No significant differences between the DES treatment group and the total control group were observed at 6 months, 1 year, or 3 years after treatment (RR 6 months: 1.06, 95% CI 0.50–2.25; 1 year: 0.97, 95% CI 0.69–1.36; 3 years: 0.89, 95% CI 0.66–1.22). No significant differences were observed between the DES group and the PTA group or the DES group and the BMS group, and no statistical heterogeneity was observed among the results. A study not included in this meta-analysis reported that paclitaxel-coated DES showed similar results for 10-year mortality compared to PTA±BMS (hazard ratio 1.05, 95% CI 0.93–1.18)^16^.

### Amputation

**Figure 4:**
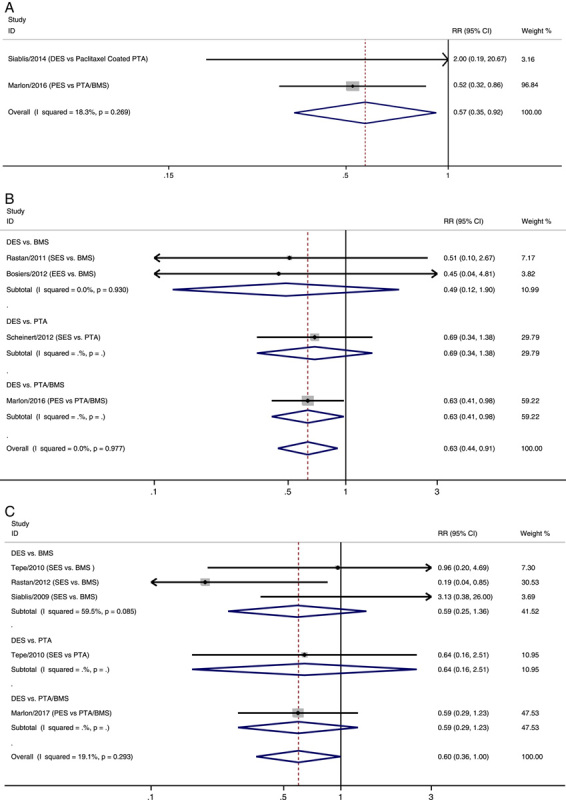
Forest plots of the estimated overall and subgroup analyses in Amputation at 6 months (A), 1 year (B) and 3 years (C). BMS, bare-metal stenting; DES, drug-eluting stent; EES, everolimus-eluting stent; PES, paclitaxel-eluting stent; PTA, percutaneous transluminal angioplasty; RR, risk ratio; SES, sirolimus-eluting stent.

Nine RCTs reported amputation outcomes (Fig. 4)^14,17–24^. DES treatment significantly reduced the rate of amputation compared with the total control group at 6 months, 1 year, and 3 years after treatment (RR 6 months: 0.57, 95% CI 0.35–0.92; 1 year: 0.63, 95% CI 0.44–0.91; 3 years: 0.60, 95% CI 0.36–1.00, *P*=0.049). No statistical heterogeneity was observed among the results. In addition, there were no significant differences between the DES group and the PTA group or the DES group and the BMS group.

### Network meta-analysis

Based on the number of RCTs that reported interest outcomes at different follow-up periods, a Bayesian analysis of the outcomes of interest was performed. We presented the total matrices (league tables) of pairwise relative treatment effects of the interest outcomes for the possible SES, EES, PES, and control group combinations (Table 2). The cumulative rank probabilities by Bayesian analysis were also summarised (Table 3).

**Table 2:** Hierarchical matrices (league tables) of pairwise relative treatment effects of the interest outcomes.

Clinical patency	6 months	PTA/BMS	—	—	—
		0.24 (0.03, 2.22)	EES	—	—
		0.59 (0.07, 4.47)	2.36 (0.12, 48.83)	PES	—
		0.24 (0.04, 1.03)	0.96 (0.05, 12.65)	0.39 (0.03, 4.58)	SES
	1 year	PTA/BMS	—	—	—
		0.21 (0.03, 1.51)	EES	—	—
		0.23 (0.08, 0.60)	1.11 (0.13, 9.87)	SES	—
Restenosis rate	6 months	PTA/BMS	—	—	—
		2.41 (0.11, 54.81)	PES	—	—
		31.58 (4.41, 307.53)	13.09 (0.39, 602.67)	SES	—
	1 year	PTA/BMS	—	—	—
		3.39 (0.79, 15.92)	EES	—	—
		3.80 (1.84, 8.87)	1.11 (0.21, 6.13)	SES	—
Target lesion revascularizations	6 months	PTA/BMS	—	—	—
		4.67 (0.35, 71.49)	EES	—	—
		6.07 (0.90, 36.24)	1.35 (0.04, 28.57)	SES	—
	1 year	PTA/BMS	—	—	—
		5.62 (0.68, 47.88)	EES	—	—
		2.72 (0.84, 11.29)	0.49 (0.04, 6.13)	SES	—
Ankle brachial index	6 months	PTA/BMS	—	—	—
		0.00 (−0.29, 0.30)	PES	—	—
		−0.03 (−0.25, 0.18)	−0.03 (−0.38, 0.31)	SES	—
	1 year	PTA/BMS	—	—	—
		−0.01 (−0.33, 0.30)	PES	—	—
		−0.07 (−0.26, 0.12)	−0.06 (−0.43, 0.31)	SES	-
All-cause death	1 year	PTA/BMS	—	—	—
		0.82 (0.24, 2.68)	EES	—	—
		1.12 (0.37, 3.31)	1.37 (0.28, 6.81)	PES	—
		1.11 (0.54, 2.32)	1.35 (0.35, 5.53)	1.00 (0.28, 3.83)	SES
	3 years	PTA/BMS	—	—	—
		1.30 (0.37, 4.76)	PES	—	—
		1.18 (0.54, 2.90)	0.89 (0.20, 4.12)	SES	—
Amputation	1 year	PTA/BMS	—	—	—
		2.50 (0.17, 74.11)	EES	—	—
		2.10 (0.71, 6.35)	0.84 (0.03, 15.06)	PES	—
		1.70 (0.62, 4.70)	0.68 (0.02, 11.76)	0.81 (0.18, 3.55)	SES

BMS, bare-metal stents; EES, everolimus-eluting stents; PES, paclitaxel-eluting stents; PTA, percutaneous transluminal angioplasty; SES, sirolimus-eluting stents.

**Table 3:** The cumulative rank probabilities in interest outcomes.

Outcome	Following time	Treatment	Rank 1	Rank 2	Rank 3	Rank 4
Clinical patency	6 months	PTA/BMS	0.00	0.03	0.27	0.69
		EES	0.45	0.35	0.14	0.07
		PES	0.10	0.21	0.47	0.22
		SES	0.46	0.41	0.12	0.02
	1 year	PTA/BMS	0.00	0.05	0.95	—
		EES	0.55	0.40	0.04	—
		SES	0.45	0.55	0.00	—
Restenosis	6 months	PTA/BMS	0.81	0.18	0.00	—
		PES	0.18	0.76	0.05	—
		SES	0.00	0.05	0.95	—
	1 year	PTA/BMS	0.96	0.04	0.00	—
		EES	0.04	0.53	0.43	—
		SES	0.00	0.43	0.57	—
Target lesion revascularizations	6 months	PTA/BMS	0.88	0.11	0.00	—
		EES	0.09	0.50	0.41	—
		SES	0.03	0.39	0.58	—
	1 year	PTA/BMS	0.92	0.08	0.00	—
		EES	0.04	0.19	0.77	—
		SES	0.04	0.73	0.23	—
Ankle brachial index	6 months	PTA/BMS	0.19	0.50	0.31	—
		PES	0.35	0.21	0.43	—
		SES	0.45	0.29	0.25	—
	1 year	PTA/BMS	0.11	0.48	0.41	—
		PES	0.34	0.21	0.45	—
		SES	0.55	0.31	0.14	—
All-cause death	1 year	PTA/BMS	0.14	0.40	0.35	0.11
		EES	0.49	0.17	0.16	0.18
		PES	0.22	0.20	0.20	0.38
		SES	0.15	0.23	0.29	0.33
	3 years	PTA/BMS	0.46	0.44	0.10	—
		PES	0.27	0.21	0.53	—
		SES	0.27	0.36	0.37	—
Amputation	1 year	PTA/BMS	0.60	0.34	0.06	0.00
		EES	0.24	0.12	0.15	0.49
		PES	0.05	0.21	0.41	0.32
		SES	0.10	0.33	0.38	0.18

BMS, bare-metal stents; EES, everolimus-eluting stents; PES, paclitaxel-eluting stents; PTA, percutaneous transluminal angioplasty; SES, sirolimus-eluting stents.

The SES group had significantly better clinical patency than that of the control group 1 year after treatment (RR 1 year: 0.23, 95% CI 0.08–0.60). There were no significant differences in the pairwise relative treatment effects of the other possible combinations. The rank probability results showed that SES ranked first in terms of the efficacy of clinical patency at 6 months after treatment (first: SES; second: EES; third: PES; fourth: PTA/BMS) and EES ranked first at 1 year after treatment (first: EES; second: SES; third: PTA/BMS).

The SES group had a significantly reduced rate of restenosis than that of the control group at 6 months and 1 year after treatment (RR 6 months: 31.58, 95% CI 4.41–307.53; 1 year: 3.80, 95% CI 1.84–8.87). There were no significant differences in the pairwise relative treatment effects of the other possible combinations. The control group had the highest restenosis rate at 6 months (first: PTA/BMS; second: PES; third: SES) and 1 year (first: PTA/BMS; second: EES; third: SES) after treatment.

The TLR network was not significantly different between any possible group combinations. The control group had the highest tendency for TLR at 6 months (first: PTA/BMS; second: EES; third: SES) and 1 year after treatment (first: PTA/BMS; second: SES; third: EES).

The ABI was not significantly different between any possible group combinations. The SES group ranked first in terms of the efficacy of ABI improvement at 6 months (first: SES; second: PTA/BMS; third: PES) and 1 year after treatment (first: SES; second: PTA/BMS; third: PES).

The all-cause mortality rate was not significantly different between any possible group combinations. The EES group had the greatest increase in all-cause mortality at 1 year after treatment (first: EES; second: PTA/BMS; third: SES; fourth: PES), and the control group had the greatest increase at 3 years after treatment (first: PTA/BMS; second: SES; third: PES).

The amputation rate was not significantly different between any possible group combinations. The control group had the highest increase in the amputation rate at 1 year after treatment (first: PTA/BMS; second: SES; third: PES; fourth: EES).

### Publication bias, sensitivity, and consistency

Publication bias was not observed for most outcome events except for the restenosis rate at 6 months after treatment (Harbord’s modified test *P*=0.068, Table 1).

Meta-trim or meta-inf operations were performed to test the sensitivity of the results. No study was required for the meta-analysis for most outcome events, and there was no change in conclusion after a single study was excluded for the meta-inf operation for ABI.

We determined that two additional studies were needed to enhance the sensitivity of the meta-analysis of clinical patency at 6 months after treatment of the DES group and the control group. However, after the meta-trim operation, the conclusions did not change for the fixed model (RR=1.166, 95% CI 1.071–1.270, *P*<0.001).

Restenosis at 3 years after treatment, all-cause mortality at 6 months after treatment, and amputation at 6 months after treatment each required one additional study to improve the sensitivity of the meta-analysis between the DES and control groups. However, after the meta-trim operation, the conclusions did not change for the fixed model (RR: 0.864, 95% CI 0.794–0.940, *P*=0.001; RR: 0.974, 95% CI 0.490–1.935, *P*=0.940; RR: 0.523, 95% CI 0.324–0.842, *P*=0.008).

The results of the Bayesian analysis random-effects and inconsistency standard deviations were similar for most outcome events, except for the restenosis rate at 6 months after treatment (random-effects standard deviation median 0.91, 95% CI 0.04–3.04; inconsistency standard deviation median 1.64, 95% CI 0.08–3.22). However, the general trend remained the same (SDC 7, Supplemental Digital Content 11, http://links.lww.com/JS9/A986).

## Discussion

The best treatment for infrapopliteal arterial disease to effectively salvage limbs has been a topic of wide concern in the field of peripheral artery disease. Although current guidelines recommend PTA as the first-line treatment, more assisted therapeutic options have been applied to the clinical treatment of infrapopliteal arterial disease, such as BMS, DCB, and DES^1,6^. There is an urgent need to systematically evaluate the differences in therapeutic effects between these treatment options. Previous meta-analyses demonstrated that the use of DES resulted in short-term benefits at 12 months compared to the use of PTA/BMS, although the benefits were not superior 3 years after treatment. However, the studies included in the previous analysis did not only include patients with infrapopliteal arterial disease, which may have confounded the results. Based on the results of the current study, DES is superior to PTA/BMS for improving clinical patency, reducing the restenosis rate, and reducing the amputation rate at 6 months, 1 year, and 3 years after treatment, suggesting that DES is more clinically effective than PTA/BMS.

As PTA is the currently recommended first-line treatment for infrapopliteal arterial disease, it is most commonly used as a control therapy in RCTs^3^. However, the difficulty of endovascular revascularisation of in-stent restenosis and restenosis of the vessel after PTA differs for patients with restenosis after the initial endovascular treatment. According to the ‘leave nothing behind’ strategy for endovascular treatment, It must be determined that the suitability of comparing the effects of DES with those of PTA or DCB for endovascular revascularisation^25^. In this meta-analysis, subgroup analyses suggested that DES is superior to BMS and PTA in improving clinical patency and reducing the target lesion revascularisation and restenosis rates at 6 months and 1 year after treatment. However, the long-term prognosis of these treatments requires further investigation as this analysis was limited by the low number of previous studies.

Although there is a dedicated DES for infrapopliteal arterial disease and a global, randomised, multicentre trial named The DES BTK Vascular Stent System vs. PTA in Subjects with Critical Limb Ischemia (SAVAL, NCT03551496) has been conducted, a DES specific for infrapopliteal arterial disease is still unavailable. Coronary DES are commonly used for the clinical treatment of infrapopliteal arterial disease. However, different anti-proliferative drug coatings of the stents may have different therapeutic effects^26,27^. Due to the limited number of published studies, the current meta-analysis included studies that were not well connected, reducing the evidential validity of the results. The DES treatment effects of the different drug coatings were ranked after consistency tests, and SES was found to be superior for improving clinical patency and reducing the restenosis rate, while EES was better at reducing TLR and amputation rates. In addition, PES were superior for improving the ABI and reducing the all-cause mortality rate. However, these results may not be sufficient. Additional RCTs are necessary to enhance the understanding of the therapeutic effects of different anti-proliferative drug coatings, provide guidance for the selection of treatment options, and develop additional DES specific for infrapopliteal arterial disease.

Based on our study, DES was superior to the control group for improving clinical patency, reducing the restenosis rate, and reducing the amputation rate, but there were no significant differences in the ABI between DES and the control group. The results of ABI can be used to stratify the severity of the peripheral arterial disease. But in the infrapopliteal artery disease, the blood flow situation below the knee is more complicated due to the presence of multiple parallel vessels, which may influence the efficacy of ABI test. In addition, the sample size of the literature included in this study was limited, and the research centres were from different countries and regions, which may increase the measurement error of ABI. For the possible follow-up clinical research, investigators should pay more attention to selecting experienced specialists to evaluate the ABI and record the ABI results at different follow-up times.

## Limitation

Due to the particularity of the Infrapopliteal Artery disease and the cost of endovascular treatment, there is less awareness and attention to this disease at the social level. The population of patients who were concerned and treated was far less than the actual population of patients. High-quality studies on this topic were scarce. Although we included only randomized controlled studies in this meta-analysis and reviewed patient population enroled in literatures to ensure that they met the inclusion criterion, there were still some limitations due to available studies. Thus, our results should be interpreted with caution. Second, some literatures did not subdivide the patients in the control group into PTA treatment and BMS treatment and did not clearly indicate the specific drug-eluting type of DES, which led to smaller sample size for outcome events in relevant subgroups. Third, WIfI system and GLASS system are proposed in the current guidelines to further evaluate the vascular lesions and prognosis of patients^6^. The literatures included in this study lacked relevant contents.

## Conclusion

The results of the current meta-analysis indicate that DES has therapeutic superiority over control therapies (PTA/BMS) for improving the clinical patency, reducing the restenosis rate, and reducing the amputation rate at 6 months, 1 year, and 3 years after treatment. In addition, the treatment effect of DES is significantly better than those of BMS or PTA at 1 year. The results of the network meta-analysis suggest that SES and EES have better clinical benefits. Further RCTs should focus on long-term outcomes, refine the treatment options of the control group to PTA or BMS separately, and subclassify the type of DES drug coatings to provide more clinical evidence for the treatment of infrapopliteal arterial disease.

## Ethical approval

No ethical approval.

## Consent

None.

## Source of funding

None.

## Author contribution

Corresponding author: Hui Zhuang, the proposal of idea, study design, correction of writing Co-first author: Yang Li and Xuwei Shen Yang Li: study design, data collections, data analysis and writing. Xuwei Shen: data collections, data analysis and writing.

## Conflicts of interest disclosure

There are no potential conflicts of interest with respect to the research, authorship, publication and/or finance of this article.

## Research registration unique identifying number (UIN)

Registry: Prospero the unique identifying number of the study: CRD42023422864.https://www.crd.york.ac.uk/prospero/display_record.php?ID=CRD42023422864.

## Guarantor

Hui Zhuang.

## Data availability statement

Data sharing is not applicable to this article as no new data were created or analysed in this study.

## Provenance and peer review

Not commissioned, externally peer-reviewed.

## Supplementary Material

SUPPLEMENTARY MATERIAL
