# Supplementary material for: Comparation of drug-eluting stents and control therapy for the treatment of infrapopliteal artery disease: a Bayesian analysis
Source: Int J Surg. 2023 Sep 14;109(12):4286–97. doi: 10.1097/JS9.0000000000000736 (PMC10720840; doi:10.1097/JS9.0000000000000736)
Supplement: SUPPLEMENTARY MATERIAL [file js9-109-4286-s002.doc]

AMSTAR 2: a critical appraisal tool for systematic reviews that include randomised or non- randomised studies of healthcare interventions, or both

| **1.** **Did** **the** **research** **questions** **and** **inclusion** **criteria** **for** **the** **review** **include** **the** **components** **of** **PICO?**   | Optional (recommended)  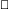 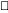  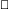 Population*√*  Yes *√* No  For Yes:  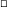 Timeframe for follow-up  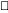 Intervention*√*  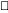 Comparator group*√*  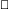 Outcome*√* | | --- |   **2.** **Did** **the** **report** **of** **the** **review** **contain** **an** **explicit** **statement** **that** **the** **review** **methods** **were** **established** **prior** **to** **the** **conduct** **of** **the** **review** **and** **did** **the** **report** **justify** **any** **significant** **deviations** **from** **the** **protocol?**   | For Partial Yes:  For Yes:  As for partial yes, plus the protocol should be registered and should also have specified:  The authors state that they had a written  protocol or guide that included ALL the  following:  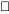 review question(s)  Yes *√* Partial Yes No  a meta-analysis/synthesis plan, if appropriate, *and √*  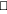  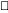 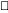 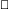  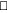 a plan for investigating causes  of heterogeneity *√*  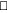 justification for any deviations  from the protocol *√*  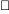 a search strategy  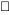 inclusion/exclusion criteria  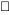 a risk of bias assessment | | --- |   **3.** **Did** **the** **review** **authors** **explain** **their** **selection** **of** **the** **study** **designs** **for** **inclusion** **in** **the** **review?**   | For Yes, the review should satisfy ONE of the following:  Yes *√* No  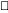 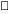  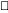 *Explanationfor* including only RCTs *√*  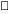 OR *Explanationfor* including only NRSI  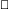 OR *Explanationfor* including both RCTs and NRSI | | --- |   **4.** **Did** **the** **review** **authors** **use** **a** **comprehensive** **literature** **search** **strategy?**   | For Yes, should also have (all the  For Partial Yes (all the following):  following):  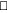 searched the reference lists /  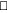 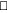 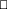  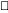 searched at least 2 databases  (relevant to research question)  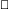 provided key word and/or  search strategy  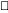 justified publication restrictions  (e.g. language)  Yes*√* Partial Yes No  bibliographies of included  studies *√*  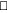 searched trial/study registries  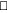 included/consulted content  experts in the field *√*  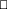 where relevant, searched for  grey literature *√*  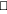 conducted search within 24  months of completion of the  review *√* | | --- |   **5.** **Did** **the** **review** **authors** **perform** **study** **selection** **in** **duplicate?** | |
| --- | --- | --- | --- | --- | --- |
| For Yes, either ONE of the following:  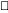 at least two reviewers independently agreed on selection of eligible studies  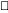 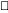  Yes *√*  No  and achieved consensus on which studies to include *√*  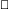 OR two reviewers selected a sample of eligible studies and achieved good  agreement (at least 80 percent), with the remainder selected by one  reviewer. |  |

AMSTAR 2: a critical appraisal tool for systematic reviews that include randomised or non- randomised studies of healthcare interventions, or both

| **6.** **Did** **the** **review** **authors** **perform** **data** **extraction** **in** **duplicate?**   | For Yes, either ONE of the following:  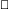 at least two reviewers achieved consensus on which data to extract from  Yes *√*  No  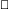 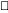  included studies *√*  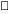 OR two reviewers extracted data from a sample of eligible studies and  achieved good agreement (at least 80 percent), with the remainder  extracted by one reviewer. | | --- |   **7.** **Did** **the** **review** **authors** **provide** **a** **list** **of** **excluded** **studies** **and** **justify** **the** **exclusions?**   | For Yes, must also have:  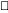 provided a list of all potentially  relevant studies that were read in full-text form but excluded from the review  Yes *√* Partial Yes No  For Partial Yes:  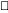 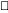 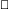  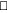 Justified the exclusion from  the review of each potentially  relevant study | | --- |   **8.** **Did** **the** **review** **authors** **describe** **the** **included** **studies** **in** **adequate** **detail?**   | For Yes, should also have ALL the  For Partial Yes (ALL the following):  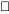 described populations 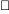 described interventions 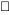 described comparators 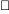 described outcomes 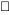 described research designs  following:  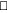 described population in detail  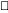 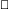 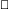  Yes *√* Partial Yes No  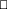 described intervention in  detail (including doses where  relevant)  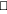 described comparator in detail  (including doses where  relevant)  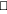 described study’s setting  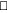 timeframe for follow-up | | --- |   **9.** **Did** **the** **review** **authors** **use** **a** **satisfactory** **technique** **for** **assessing** **the** **risk** **of** **bias** **(RoB)** **in** **individual** **studies** **that** **were** **included** **in** **the** **review?**   | **RCTs**  For Partial Yes, must have assessed RoB  For Yes, must also have assessed RoB  from:  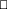 allocation sequence that was 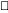 Yes *√*  not truly random, *and* 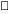 Partial Yes  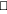 selection of the reported result 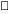 No  from among multiple 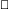 Includes only  measurements or analyses of a NRSI  specified outcome  from  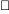 unconcealed allocation, *and*  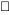 lack of blinding of patients and  assessors when assessing  outcomes (unnecessary for  objective outcomes such as all-  cause mortality) | | --- | | **NRSI**  For Partial Yes, must have assessed  For Yes, must also have assessed RoB:  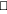 methods used to ascertain 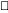 Yes  exposures and outcomes, *and* 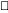 Partial Yes  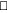 selection of the reported result 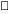 No  from among multiple 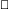 Includes only  measurements or analyses of a RCTs  specified outcome  RoB:  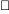 from confounding, *and*  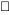 from selection bias |   **10.** **Did** **the** **review** **authors** **report** **on** **the** **sources** **of** **funding** **for** **the** **studies** **included** **in** **the** **review?** | | | |
| --- | --- | --- | --- | --- | --- | --- | --- | --- |
|  | For Yes  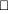 Must have reported on the sources of funding for individual studies included  in the review. Note: Reporting that the reviewers looked for this information but it was not reported by study authors also qualifies | 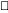 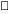 | Yes*√*  No |

AMSTAR 2: a critical appraisal tool for systematic reviews that include randomised or non- randomised studies of healthcare interventions, or both

| **11.** **If** **meta-analysis** **was** **performed** **did** **the** **review** **authors** **use** **appropriate** **methods** **for** **statistical**  **combination** **of** **results?**   | **RCTs**  For Yes:  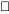 The authors justified combining the data in a meta-analysis  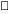 Yes *√*  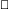 No 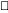 No meta-analysis  conducted  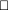 AND they used an appropriate weighted technique to combine  study results and adjusted for heterogeneity if present.  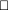 AND investigated the causes of any heterogeneity | | --- | | **For** **NRSI**  For Yes:  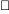 The authors justified combining the data in a meta-analysis  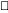 Yes  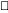 No 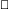 No meta-analysis  conducted  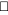 AND they used an appropriate weighted technique to combine  study results, adjusting for heterogeneity if present  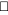 AND they statistically combined effect estimates from NRSI that  were adjusted for confounding, rather than combining raw data,  or justified combining raw data when adjusted effect estimates  were not available  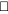 AND they reported separate summary estimates for RCTs and  NRSI separately when both were included in the review |   **12.** **If** **meta-analysis** **was** **performed,** **did** **the** **review** **authors** **assess** **the** **potential** **impact** **of** **RoB** **in**  **individual** **studies** **on** **the** **results** **of** **the** **meta-analysis** **or** **other** **evidence** **synthesis?**   | For Yes:  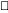 included only low risk of bias RCTs *√*  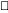 Yes*√*  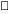 No 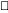 No meta-analysis  conducted  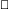 OR, if the pooled estimate was based on RCTs and/or NRSI at variable  RoB, the authors performed analyses to investigate possible impact of  RoB on summary estimates of effect. | | --- |   **13.** **Did** **the** **review** **authors** **account** **for** **RoB** **in** **individual** **studies** **when** **interpreting/** **discussing** **the** **results** **of** **the** **review?**   | For Yes:  Yes*√* No  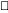 included only low risk of bias RCTs *√*  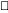 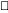  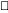 OR, if RCTs with moderate or high RoB, or NRSI were included the  review provided a discussion of the likely impact of RoB on the results | | --- |   **14.** **Did** **the** **review** **authors** **provide** **a** **satisfactory** **explanation** **for,** **and** **discussion** **of,** **any** **heterogeneity** **observed** **in** **the** **results** **of** **the** **review?**   | For Yes:  There was no significant heterogeneity in the results  OR if heterogeneity was present the authors performed an investigation of    Yes*√* No  sources of any heterogeneity in the results and discussed the impact of this  on the results of the review*√* | | --- |   **15.** **If** **they** **performed** **quantitative** **synthesis** **did** **the** **review** **authors** **carry** **out** **an** **adequate** **investigation** **of** **publication** **bias** **(small** **study** **bias)** **and** **discuss** **its** **likely** **impact** **on** **the** **results** **of** **the** **review?** | |
| --- | --- | --- | --- | --- | --- | --- |
| For Yes:  performed graphical or statistical tests for publication bias and discussed  Yes*√*  No No meta-analysis  conducted  the likelihood and magnitude of impact of publication bias |  |

AMSTAR 2: a critical appraisal tool for systematic reviews that include randomised or non- randomised studies of healthcare interventions, or both

| **16.** **Did** **the** **review** **authors** **report** **any** **potential** **sources** **of** **conflict** **of** **interest,** **including** **any** **funding** **they** **received** **for** **conducting** **the** **review?** | |
| --- | --- |
| For Yes:  The authors reported no competing interests OR *√* Yes*√*  The authors described their funding sources and how they managed No  potential conflicts of interest |  |

**To** **cite** **this** **tool:** Shea BJ, Reeves BC, Wells G, Thuku M, Hamel C, Moran J, Moher D, Tugwell P, Welch V, Kristjansson E, Henry DA. AMSTAR 2: a critical appraisal tool for systematic reviews that include randomised or non-randomised studies of healthcare interventions, or both. BMJ. 2017 Sep 21;358:j4008.
