# Supplementary material for: Comparation of drug-eluting stents and control therapy for the treatment of infrapopliteal artery disease: a Bayesian analysis
Source: Int J Surg. 2023 Sep 14;109(12):4286–97. doi: 10.1097/JS9.0000000000000736 (PMC10720840; doi:10.1097/JS9.0000000000000736)
Supplement: SUPPLEMENTARY MATERIAL [file js9-109-4286-s003.docx]

The supplement digital content 2. The search strategy performed for PubMed

1.

“Clinical trial”[Mesh]

2.

“Randomized Controlled Trial”[Mesh]

3.

“Clinical trial”[TW]

4.

“Randomized “[TW]

5.

“Peripheral Arterial Disease”[Mesh]

6.

peripheral occlusive artery disease[All Fields]

7.

“Peripheral Arterial Disease”[TW]

8.

“Peripheral Artery Disease”[TW]

9.

“Peripheral Arterial Occlusive Disease”[TW]

10.

“Intermittent Claudication”[Mesh]

11.

“Intermittent Claudication”[TW]

12.

“Ischemia”[Mesh]

13.

“Critical limb ischemia”[TW]

14.

“Amputation”[Mesh]

15.

“Infrapopliteal”[TW]

16.

“below-the-knee”[TW]

17.

“percutaneous”[TW]

18.

“Stents”[Mesh]

19.

“Bare”[TW]

20.

“Bare-metal”[TW]

21.

“Metal”[TW]

22.

“Stent”[TW]

23.

“Drug-Eluting Stents”[Mesh]

24.

“Drug-Coated Stents”[Mesh]

25.

“Drug-eluting”[TW]

26.

“Drug-coated”[TW]

27.

“Paclitaxel-coated”[TW]

28.

“Paclitaxel”[Mesh]

29.

“Sirolimus-Eluted”[TW]

30.

“Sirolimus”[TW]

31.

“Everolimus”[TW]

32.

“Everolimus-Eluted”[TW]

Search String

(#1 OR #2 OR #3 OR #4) AND

(#5 OR #6 OR #7 OR #8 OR #9 OR #10 OR #11 OR #12 OR #13 OR #14 OR #15 OR #16) AND

(#17 OR #18 OR #19 OR #20 OR #21 OR #22 OR #23 OR #24 OR #25 OR #26 OR #27 OR #28 OR #29 OR #30 OR #31 OR #32)
