# Supplementary material for: Comparation of drug-eluting stents and control therapy for the treatment of infrapopliteal artery disease: a Bayesian analysis
Source: Int J Surg. 2023 Sep 14;109(12):4286–97. doi: 10.1097/JS9.0000000000000736 (PMC10720840; doi:10.1097/JS9.0000000000000736)
Supplement: SUPPLEMENTARY MATERIAL [file js9-109-4286-s004.docx]

The supplement digital content 3. Risk of bias of studies included in meta-analysis

| **Reference (year)** | **Random sequence generation** | **Allocation concealment** | **Blinding of participants and personnel** | **Blinding of outcome assessment** | **Incomplete outcome data** | **Selective reporting** | **Other bias** |
| --- | --- | --- | --- | --- | --- | --- | --- |
| Aleksander/2008 | Low | Low | Low | Low | Low | Unclear | Low |
| Siablis/2009 | Unclear | Unclear | Unclear | Low | Low | Unclear | Low |
| Tepe/2010 | Unclear | Unclear | Unclear | Low | Low | Low | Low |
| Rastan/2011 | Low | Low | Low | Low | Low | Low | Low |
| Bosiers/2012 | Low | Low | Low | Low | Low | Low | Low |
| Rastan/2012 | Low | Low | Low | Low | Low | Low | Low |
| Scheinert/2012 | Low | Low | Low | Low | Low | Low | Low |
| Siablis/2014 | Low | Low | Low | Low | Low | Low | Low |
| He Tao/2015 | Low | Low | Unclear | Unclear | Low | Unclear | Low |
| Marlon/2016 | Low | Low | Low | Low | Low | Low | Low |
| Marlon/2017 | Low | Low | Low | Low | Low | Low | Low |
| Marlon/2020 | Low | Low | Low | Low | Low | Low | Low |
