# Supplementary material for: Comparation of drug-eluting stents and control therapy for the treatment of infrapopliteal artery disease: a Bayesian analysis
Source: Int J Surg. 2023 Sep 14;109(12):4286–97. doi: 10.1097/JS9.0000000000000736 (PMC10720840; doi:10.1097/JS9.0000000000000736)
Supplement: SUPPLEMENTARY MATERIAL [file js9-109-4286-s005.docx]

The supplement digital content 4. Detailed baseline characteristics synthesis of the 12 studies pooled in the systematic review and meta-analysis

| Auther/Year/Country | Trial or registration | treatment and control | patients (DES/ control) | the Rutherford scale | lesion length (mm) | mean Ref vessel diameter (mm) | Follow up period |
| --- | --- | --- | --- | --- | --- | --- | --- |
| Aleksander/2008/Poland | non | SES vs. BMS | 50(25/25) | Rutherford 3: DES 16/25control18/25; Rutherford 4: DES6/25control4/25; Rutherford 5: DES3/25control3/25; | DES: 17.4±3.2 control: 18.2±3.5 | DES: 2.69±1.8 control: 2.68±1.6 | 6 months |
| Tepe/2010/Germany | The BELOW Study | SES vs. BMS vs. PTA | 44(14/16/14) | Rutherford 5,6 | SES: 27±25 BMS: 35±17 PTA: 31±17 | SES: 2.9±0.22 BMS: 2.8±0.25 PTA: 2.9±0.25 | 2,6 months; telephone interview (2-4 years) |
| Rastan/2011/Germany | NCT00664963 | SES vs. BMS | 161(82/79) | Rutherford–Becker class (RC) of 3 to 5. | SES: 30±8 BMS: 31±9 | SES: 3±0.4 BMS: 3±0.4 | 6,12 months |
| Bosiers/2012/multicenter European | The DESTINY trial; NTC00510393 | EES vs. BMS | 140(74/66) | Rutherford 4: EES 37/74 BMS 26/66 Rutherford 5: EES 37/74 BMS 40/66 | EES: 15.9±10.2 BMS: 18.9±10.0 | EES: 3.00±0.84 BMS: 2.91±0.72 | 12 months |
| Rastan/2012/Germany | NCT00664963 | SES vs. BMS | 161(82/79) | Rutherford–Becker class of 3 to 5. | SES: 30±8 BMS: 31±9 | SES: 3±0.4 BMS: 3±0.4 | 3 years |
| Scheinert/2012/in 9 European countries | The ACHILLES trial | SES vs. PTA | 200(99/101) | Rutherford class 3 to 5 | SES: 26.9±20.9 PTA: 26.8±21.3 | SES: 2.6±0.5 PTA: 2.6±0.6 | 12 months |
| Siablis/2014/Greece | The IDEAS trial; NCT01517997 | DES vs. Paclitaxel-PTA | 50(25/25) | Rutherford classes 3 to 6 | DES: 127±46.5 PCB: 148±56.7 | non | 6 months |
| Marlon/2016/ in 3 major vascular centers in the Netherlands | The PADI trial; NCT00471289 | PES vs. PTA±BMS | 137(73/64) | Rutherford 4: DES 10/74 PTA±BMS 8/66 Rutherford 5: DES 48/74 PTA±BMS 46/66 Rutherford 6: DES 16/74 PTA±BMS 12/66 | DES: 21.1±19.3 PTA±BMS: 23.1±21.8 | DES: 2.9±0.7 PTA±BMS: 2.9±0.6 | 6 months |
| Marlon/2017/in 3 major vascular centers in the Netherlands | The PADI trial; NCT00471289 | PES vs. PTA±BMS | 137(73/64) | Rutherford 4: DES 10/74 PTA±BMS 8/66 Rutherford 5: DES 48/74 PTA±BMS 46/66 Rutherford 6: DES 16/74 PTA±BMS 12/66 | DES: 21.1±19.3 PTA±BMS: 23.1±21.8 | DES: 2.9±0.7 PTA±BMS: 2.9±0.6 | 3,5 years |
| Marlon/2020/in 3 major vascular centers in the Netherlands | The PADI trial; NCT00471289 | PES vs. PTA±BMS | 137(73/64) | Rutherford 4: DES 10/74 PTA±BMS 8/66 Rutherford 5: DES 48/74 PTA±BMS 46/66 Rutherford 6: DES 16/74 PTA±BMS 12/66 | DES: 21.1±19.3 PTA±BMS: 23.1±21.8 | DES: 2.9±0.7 PTA±BMS: 2.9±0.6 | 10 years |
| Siablis/2009/Greece | non | SES vs. BMS | 103(62/41) | Rutherford 4: SES 26/62 BMS 15/41 Rutherford 5: SES 26/62 BMS 16/41 Rutherford 6: SES 10/62 BMS 10/41 | SES: 54.97±55.85 BMS: 45.18±41.94 | non | 3 years |
| He Tao/2015/China | non | SES vs. PTA | 68(34/34) | Rutherford 3-6 | non | non | 6,12 months |

Table 1 (*continued*). Detailed baseline characteristics synthesis of the 12 studies pooled in the systematic review and meta-analysis

| Auther/Year/Country | Lumen diameter (mm) | Male(%) | Smoking(%) | Cardiac disease | Diabetes | Hyperlipidemia |
| --- | --- | --- | --- | --- | --- | --- |
| Aleksander/2008/Poland | DES: 2.69±1.8 control: 2.68±1.6, p=ns | DES: 14/25 control: 15/25, p=ns | DES: 12/25 control: 10/25, p=ns | DES: 9/25 control: 12/25, p=ns | DES: 10/25 control: 10/25, p=ns | DES: 8/25 control: 10/25, p=ns |
| Tepe/2010/Germany | non | SES: 7/14 BMS: 9/16 PTA: 9/14 | SES: 2/14 BMS: 0/16 PTA: 2/14 | non | SES: 7/14 BMS: 8/16 PTA: 14/14 | SES: 4/14 BMS: 5/16 PTA: 8/14 |
| Rastan/2011/Germany | non | SES: 56/82 BMS: 51/79, p=ns | SES: 23/82 BMS: 23/79, p=ns | non | SES: 47/82 BMS: 40/79, p=ns | SES: 63/82 BMS: 61/79, p=ns |
| Bosiers/2012/multicenter European | non | EES: 45/74 BMS: 44/66, p=ns | EES: 23/74 BMS: 22/66, p=ns | non | EES: 44/74 BMS: 33/66, p=ns | EES: 28/74 BMS: 25/66, p=ns |
| Rastan/2012/Germany | non | SES: 56/82 BMS: 51/79, p=ns | SES: 23/82 BMS: 23/79, p=ns | non | SES: 47/82 BMS: 40/79, p=ns | SES: 63/82 BMS: 61/79, p=ns |
| Scheinert/2012/in 9 European countries | non | SES: 67/99 PTA: 76/101, p=ns | SES: 38/99 PTA: 27/101, p=ns | SES: 45/99 PTA: 45/101, p=ns | SES: 64/99 PTA: 65/101, p=ns | SES: 77/99 PTA: 69/101, p=ns |
| Siablis/2014/Greece | non | DES: 18/25 PCB: 20/25, p=ns | DES: 6/25 PCB: 9/25, p=ns | DES: 8/25 PCB: 6/25, p=ns | DES: 16/25 PCB: 19/25, p=ns | DES: 13/25 PCB: 10/25, p=ns |
| Marlon/2016/ in 3 major vascular centers in the Netherlands | non | DES: 49/73 PTA±BMS: 47/64, p=ns | DES: 34/73 PTA±BMS: 29/64, p=ns | DES: 27/73 PTA±BMS: 25/64, p=ns | DES: 44/73 PTA±BMS: 43/64, p=ns | non |
| Marlon/2017/in 3 major vascular centers in the Netherlands | non | DES: 49/73 PTA±BMS: 47/64, p=ns | DES: 34/73 PTA±BMS: 29/64, p=ns | DES: 27/73 PTA±BMS: 25/64, p=ns | DES: 44/73 PTA±BMS: 43/64, p=ns | non |
| Marlon/2020/in 3 major vascular centers in the Netherlands | non | DES: 49/73 PTA±BMS: 47/64, p=ns | DES: 34/73 PTA±BMS: 29/64, p=ns | DES: 27/73 PTA±BMS: 25/64, p=ns | DES: 44/73 PTA±BMS: 43/64, p=ns | non |
| Siablis/2009/Greece | non | SES: 44/62 BMS: 37/41, p=0.009 | SES: 22/62 BMS: 21/41, p=ns | SES: 31/62 BMS: 19/41, p=ns | SES: 54/62 BMS: 31/41, p=ns | SES: 47/62 BMS: 28/41, p=ns |
| He Tao/2015/China | non | SES: 22/34 PTA: 24/34, p=ns | non | SES: 16/34 PTA: 15/34, p=ns | SES: 15/34 PTA: 16/34, p=ns | SES: 12/34 PTA: 13/34, p=ns |

Table 1 (*continued*). Detailed baseline characteristics synthesis of the 12 studies pooled in the systematic review and meta-analysis

| Auther/Year/Country | Clinical patency | Restenosis rate (≥50%) | Target lesion revascularization | ABI | all-cause death | minor and major amputation |
| --- | --- | --- | --- | --- | --- | --- |
| Aleksander/2008/Poland | DES: 24/25 control: 19/25 | DES: 4/25 control: 19/25 | DES: 3/25 control: 14/25 | befoe: SES 0.51±0.07 BMS 0.52±0.07; after: SES 0.50±0.034 BMS 0.61±0.03; | non | non |
| Tepe/2010/Germany | non | SES: 1/14(1 occlusion) BMS: 6/9(1 occlusion) PTA: 7/9(2 occlusion) | SES: 1/14 BMS: 2/9 PTA: 1/9 | non | SES: 1/14 BMS: 3/14 PTA: 1/9 | SES: 3/14 BMS: 2/9 PTA: 3/9 |
| Rastan/2011/Germany | 1 year: SES 50/62 BMS 35/63; 6 months: SES 50/64 BMS 35/67 | 1 year: SES 12/62 BMS 28/63 | 1 year: SES 6/62 BMS 11/63 | befoe: SES 0.47±0.18 BMS 0.49±0.14; after: SES 0.86±0.15 BMS 0.83±0.19; | 1year: SES 14/82 BMS 11/79 | 1 year: SES 2/62 BMS 4/63 |
| Bosiers/2012/multicenter European | 1 year: EES 66/78 BMS 41/76; 6 months: EES 74/78 BMS 63/76 | 1 year: EES 17/75 BMS 36/73; | 1 year: EES 7/78 BMS 26/76; 6 months: EES 3/78 BMS 11/76 | non | 1 year: EES 13/74 BMS 10/66; | 1 year: EES 1/74 BMS 2/66; |
| Rastan/2012/Germany | non | non | 3year: SES 7/82 BMS 15/79 | non | 3year: SES 17/82 BMS 18/79 | 3year: SES 2/82 BMS 10/79 |
| Scheinert/2012/in 9 European countries | 1 year: SES 54/72 PTA 44/77 | 1 year: SES 15/67 PTA 31/74 | 1 year: SES 8/80 PTA 14/85 | non | 1 year: SES 10/99 PTA 12/101 | 1 year: SES 11/80 PTA 17/85 |
| Siablis/2014/Greece | non | 6months DES: 7/25 PCB: 11/19 | 6months DES: 2/26 PCB: 3/22 | non | DES: 3/25 PCB: 2/25 | DES: 2/25 PCB: 1/25 |
| Marlon/2016/ in 3 major vascular centers in the Netherlands | 6 months: DES: 47/98 PTA±BMS: 27/77 | 6 months: DES: 15/98 PTA±BMS: 23/77 | non | at basement: DES 0.75(0.66-0.84) PTA±BMS 0.74(0.66-0.82); 6months: DES 0.85(0.76-0.94) PTA±BMS 0.83(0.73-0.93); 1year: DES 0.94(0.80-1.09) PTA±BMS 0.91(0.79-1.03); | 6 months: DES: 10/73 PTA±BMS: 9/64; 1 year: DES: 17/73 PTA±BMS: 16/64; | 6 months: DES: 17/74 PTA±BMS: 29/66; 1 year: DES: 22/74 PTA±BMS: 31/66; |
| Marlon/2017/in 3 major vascular centers in the Netherlands | 3years: DES: 20/53 PTA±BMS: 8/39; 5years: DES: 5/43 PTA±BMS: 3/35; | 3years: DES: 4/53 PTA±BMS: 3/39; 5years: DES: 2/43 PTA±BMS: 1/35; | 3years: DES: 4/73 PTA±BMS: 4/64; 5years: DES: 3/73 PTA±BMS: 1/64; | non | 3years: DES: 22/73 PTA±BMS: 23/64; 5years: DES: 32/73 PTA±BMS: 31/64; | 3years: DES: 10/74 PTA±BMS: 15/66; 5years: DES: 11/74 PTA±BMS: 17/66; |
| Marlon/2020/in 3 major vascular centers in the Netherlands | non | non | non | non | 10years: DES: 59/73 PTA±BMS: 50/64; | non |
| Siablis/2009/Greece | 3years: SES: 50/153 BMS: 13/77; HR 4.81 2.91–7.94; 1year: SES: 114/153 BMS: 23/77; | 3years: SES: 127/153 BMS: 74/77; HR 0.45 0.29–0.68; 1years: SES: 58/153 BMS: 56/77; | non | non | 3years: SES: 20/62 BMS: 12/41; HR 1.84 0.70–4.80; 1years: SES: 4/62 BMS: 5/41; | 3years: SES: 5/75 BMS: 1/47; |
| He Tao/2015/China | 1year SES: 32/34 PTA: 23/34 | 1year SES: 2/34 PTA: 11/34 | 1year SES: 2/34 PTA: 11/34 | basement SES: 0.35±0.06 PTA: 0.33±0.08; 6months SES: 0.85±0.04 PTA: 0.62±0.03; 1year SES: 0.80±0.04 PTA: 0.53±0.02; | non | non |

^[[1]](#footnote-1)^

1. SES: sirolimus-eluting stents; EES: everolimus-eluting stents; PES: paclitaxel-eluting stents; BMS: bare-metal stents; PTA: percutaneous transluminal angioplasty [↑](#footnote-ref-1)
