# Supplementary material for: Comparation of drug-eluting stents and control therapy for the treatment of infrapopliteal artery disease: a Bayesian analysis
Source: Int J Surg. 2023 Sep 14;109(12):4286–97. doi: 10.1097/JS9.0000000000000736 (PMC10720840; doi:10.1097/JS9.0000000000000736)
Supplement: SUPPLEMENTARY MATERIAL [file js9-109-4286-s006.docx]

The supplement digital content 5. The patient selection criteria in enrolled literatures.

Aleksander/2008/Poland

Inclusion criteria: Patients were included in the study when they had a primary stenosis in at least one of the crural arteries, minimum 60% in length, 0.5–3.0 cm, with the diameter of the vessel in the unchanged site 2.0–3.5 mm, as well as successful passage with the guidewire through the stenosis.

Exclusion criteria:

1. Impaired common aortal-iliac or femoral flow defined by the presence of hemodynamically significant atherosclerotic lesions in the aorta, common iliac, or crural arteries.
2. the following criteria excluded stenting: Buerger’s disease; aneurysm of the treated vessel; earlier percutaneous treatment of arteries on the side of cured lesion; the presence of aortal, iliac, or femoral vascular prosthesis; severe calcification or tortuosity of the vessel that would make the stent placement difficult.
3. Other exclusion criteria were: thrombophlebitis; severe renal insufficiency requiring dialysis; liver failure; therapy with immunosuppressants; hemorrhagic stroke in the past 3 months; an allergy to acetylsalicylic acid, clopidogrel, heparin, or contrast medium; suspicion or confirmed active infection; disorder of coagulation; the number of platelets <150,000/mm3 or >450,000/mm3; a procedure on coronary vessels within 30 days before the inclusion in the above-mentioned study or planned procedure on coronary vessels during 30 days after the inclusion; life expectancy less than 2 years; pregnancy. Patients who might have been pregnant needed to have had a negative result of a pregnancy test in a period no longer than 3 days before the procedure.

Tepe/2010/Germany

Inclusion criteria: Rutherford stage V-VI with current ulcers on the basis of arterial disease, a patent vessel to the distal lower leg and an index lesion of maximum 5 cm in length.

Exclusion criteria: Not mention.

Rastan/2011/Germany

Inclusion criteria:

1. Patients were eligible for the study if they were at least 21 years old, were not pregnant, and suffered from peripheral artery disease with a Rutherford–Becker class (RC) of 3 to 5. Patients with lifestyle-limiting claudication RC 2 could also be included after successful intervention of TASC A (single stenosis less than 3 cm of the superficial femoral artery or popliteal artery) femoro-popliteal lesions to improve run-off status.
2. Angiographic eligibility criteria were the presence of a single primary target lesion in a native IPA that was 2.5–3.5 mm in diameter and that did not exceed 45 mm in length to assure complete lesion coverage by the treatment with a maximum of two stents with a stent length of 25 mm; diameter stenosis of at least over 70%, as estimated by duplex-ultrasound (DU) and visually on angiography.

Exclusion criteria: A visible thrombus within target lesion, known systemic coagulopathy, Buerger’s disease, acute limb ischemia, and life expectancy less than 1 year, or an intolerance of aspirin, clopidogrel, and heparin.

Bosiers/2012/multicenter European

Inclusion criteria: Patients were eligible for enrollment in the DESTINY trial if they had symptomatic PAD due to a maximum of two focal de novo atherosclerotic target lesions in one or more infrapopliteal vessels. Lesions with ≥50% diameter stenosis were considered for the trial when their length was ≤40 mm and they arose in target vessels with diameters of 2.0 to 3.5 mm. Lesions that arose in arteries located in the upper half of the lower leg were considered “proximal,” and those arising in the lower half of the lower leg were considered “distal.” Patients with significant inflow stenosis were eligible for inclusion if the stenosis had been successfully treated without complication. All patients were required to have at least one patent tibial artery that provided in-line circulation to the foot. At least single-vessel outflow to the foot (distal to the target lesion) was an entry criterion for the study.

Exclusion criteria: Not mention.

Rastan/2012/Germany

As described above.

Scheinert/2012/in 9 European countries

Inclusion criteria:

1. Subject must be ≥ 18 and ≤ 85 years old.
2. Female of childbearing potential must have a negative pregnancy test within 10 days prior to index procedure and utilize reliable birth control until completion of the 12-month angiographic follow-up evaluation.
3. Clinical diagnosis of symptomatic critical limb ischemia as defined by Rutherford 3, 4 or 5.
4. Single treatment of de novo or restenotic (after PTA only) lesion(s) in the tibioperoneal trunk, anterior and/or posterior tibial and/or peroneal artery.
5. A maximum of 2 vessels in 1 limb may be treated in the study, each vessel for only 1 target lesion, resulting in at single risk target lesion(s); In case 1 target lesion is located in the tibioperoneal trunk; the 2nd target lesion (if applicable) can only be located in the anterior tibial artery; Additional non-target lesion(s) in remaining non-target vessel(s) can be treated at the physician’s discretion by means of balloon dilatation (± bail out stenting).
6. The sum of the total length of both target lesions can be maximum 120 mm.
7. In total a maximum of 4 stents may be implanted to fully cover the maximum of 2 target lesions per subject.
8. Target vessel is ≥ 2.5 and ≤ 3.5 mm in diameter (visual estimate).
9. Target lesion stenosis is > 70% diameter stenosis (visual estimate).
10. Guidewire must be across the first (if applicable) target lesion and located intraluminally within the distal vessel before the study randomization.
11. Willing to comply with the specified follow up evaluation.
12. Written informed consent prior to any study procedures.

Exclusion criteria:

1. Significant (>50%) stenoses distal to the target lesion that might require revascularization or impede run-off.
2. Angiographic evidence of thrombus within the target vessel.
3. Thrombolysis within 72 hours prior to the index procedure.
4. Lesions not suitable for stenting.
5. Lesions (>75% stenosis) in the common or external iliac, common or superficial femoral (SFA) and popliteal artery. However, intervention in TASC A and B lesions (max 15 cm), to restore adequate blood flow, in the same index procedure is allowed. This intervention must be prior to treatment of the study lesion(s) and successful.
6. Lesions located at the bifurcation requiring treatment of both branches (1 in main branch and 1 in side branch).
7. Required stent placement across or within 1 cm of the knee joint or in an artery subject to external compression or in an artery directly subject to movement of the ankle or knee joint.
8. Prior stenting within the target vessel(s).
9. Aneurysm in the SFA or popliteal artery.
10. Requiring popliteal arterial access.
11. Concomitant hepatic insufficiency, thrombophlebitis, deep venous thrombosis, coagulation disorder or receiving immunosuppressive therapy.
12. Recent MI or stroke or coronary intervention (<30 days prior to the index procedure).
13. Life expectancy < 12 months.
14. Known or suspected active infection at the time of the index procedure (excluding an infection of a lower extremity wound of the target limb).
15. Impaired renal function (Cr > 2.5 mg/dl).
16. Known or suspected allergies or contra-indications to anti-platelet agents, heparin, stainless-steel or contrast media.
17. Current prescription for Coumadin/warfarin which in the investigator’s opinion interferes with the subject’s participation.
18. Any significant medical condition which in the investigator’s opinion may interfere with the subject’s optimal participation.
19. Participation in another investigational drug/device study that has not completed its primary endpoint or that clinically interferes with the endpoints of this study.

Siablis/2014/Greece

Inclusion criteria:

1. Angiographically documented infrapopliteal arterial occlusive disease.
2. Rutherford classification ≥3.
3. Single lesion ≥70 mm (maximum 2 arteries).
4. Life expectancy ≥1 yr.
5. Age 18 to 90 yrs.

Exclusion criteria:

1. Comorbidities excluding the patient from angioplasty procedure.
2. Allergic reaction to contrast media.
3. Known hypersensitivity to the devices’ drug agents.
4. Below-the-ankle arterial occlusive disease.
5. Known contraindication to aspirin and/or clopidogrel.

Marlon/2016/ in 3 major vascular centers in the Netherlands

Inclusion criteria:

1. Age>18years.
2. If female patient with child-bearing potential, patient may not be pregnant at the study entry and must utilize reliable birth control for the duration of her participation in the study.
3. Patient is willing and able to comply with the specified follow-up evaluation.
4. Critical limb ischemia, defined as Rutherford category 4 (ischemic rest pain), 5 (minor tissue loss), or 6 (major tissue loss).
5. Stenosis (>50% luminal loss) or occlusion of an infrapopliteal artery, including the tibiofibular trunk, the anterior tibial artery, the posterior tibial artery, and the peroneal artery.
6. Target lesion length ≤90 mm.
7. Artery to be treated with a diameter ≥2 mm and ≤6mm.
8. Patent common iliac, external iliac, superficial femoral and popliteal artery on the ipsilateral side prior to randomization, possibly after treatment during the same session.
9. At least 1 patent crural (anteriortibial, posterior tibial, or peroneal) artery with expected unobstructed runoff to ankle level after treatment.

Exclusion criteria:

1. Acute limb ischemia
2. Previous amputation of affected limb at or above ankle level.
3. Subacute limb ischemia which requires thrombolysis as first treatment modality.
4. Active bleeding or bleeding diathesis.
5. Recent (≤3 months) hemorrhagic stroke or any other CNS abnormality with increased risk of hemorrhage, such as intracranial neoplasm, arteriovenous malformation, intracranial aneurysm, or aneurysm repair.
6. Gastrointestinal or genitourinary bleeding of clinical significance within the previous 6 weeks before treatment.
7. Aneurysm in common femoral, superficial femoral, or popliteal artery on the ipsilateral side.
8. Surgical revascularization involving the same limb within 30 days prior to the index procedure or planned surgical revascularization of the same limb within 30 days of the index procedure.
9. Previous implanted stent at the index site.
10. Life expectancy of less than 6 months or other factors making clinical follow-up difficult.
11. Known allergy to acetylsalicylic acid (aspirin), clopidogrel, heparin, or paclitaxel.
12. Known allergy to contrast media.
13. Known heparin-induced thrombocytopenia (HIT type 2).
14. Patient unable or unwilling to tolerate anticoagulant, anti-platelet therapy or contrast media.
15. Creatinine clearance 20 mL/minute (as derived from Cockroft-Gault formula).
16. Severely calcified lesions with expected resistance to stenting.
17. Poor inflow due to ipsilateral stenoses or occlusions of the iliac or femoropopliteal arteries that cannot be treated during the same session.
18. Significant vessel tortuosity or other parameters prohibiting access to the lesions and/or delivery of the stent.
19. Patients without (expected) distal runoff to the index site.

Marlon/2017/in 3 major vascular centers in the Netherlands

As described above.

Marlon/2020/in 3 major vascular centers in the Netherlands

As described above.

Siablis/2009/Greece

Inclusion criteria:

1. CLI symptoms (Rutherfordcategories 4–6).
2. DSA documentation of infrapopliteal obstructive arterial disease.
3. Bail-out stenting after suboptimal and/or complicated below-knee angioplasty.
4. Reference diameter of native tibial vessel less than 4 mm.

Exclusion criteria:

1. History of severe contrast allergy/hypersensitivity.
2. Hypersensitivity to aspirin and/or clopidogrel.
3. Systemic coagulopathy or hypercoagulation disorders.
4. Acute limb ischemia.
5. Buerger disease.
6. Deep vein thrombosis.
7. Bifurcation and/or trifurcation lesions.
8. Previous use of other drug-eluting stent (not SES).
9. Stenting indications after suboptimal and/or complicated balloon angioplasty, Elastic recoil, Flow-limiting dissection, Residual stenosis more than 30%.

He Tao/2015/China

Inclusion criteria:

1. consent to accept balloon dilation or drug-eluting stent implantation of the diseased artery.
2. proximal main artery disease of below-the-knee artery, namely, tibiofibular artery, anterior tibial artery, posterior tibial artery or peroneal artery disease, with or without superficial femoral artery and iliac artery disease.
3. The distal outflow tract of the diseased artery was patent, and at least one of the three branches below the knee was completely patent.
4. Physical condition meets the needs of surgery.

Exclusion criteria: Not mention.
