# Supplementary material for: Comparation of drug-eluting stents and control therapy for the treatment of infrapopliteal artery disease: a Bayesian analysis
Source: Int J Surg. 2023 Sep 14;109(12):4286–97. doi: 10.1097/JS9.0000000000000736 (PMC10720840; doi:10.1097/JS9.0000000000000736)
Supplement: SUPPLEMENTARY MATERIAL [file js9-109-4286-s007.docx]

The supplement digital content 6. The anticoagulant and antiaggregant use and the information of stents.

| Auther/Year/Country | Preproceduce | Postproceduce | Stents information |
| --- | --- | --- | --- |
| Aleksander/2008/Poland | Three days before the procedure, patients received oral acetylsalicylic acid 100 mg/day and/or clopidogrel 75 mg/ day; During the procedure, 5,000 IU of heparin was given intra-arterially | In the first 24 h after the procedure, two separate 40-mg doses of low molecular weight heparin were administered subcutaneously: Afterwards acetylsalicylic acid 100 mg/day and clopidogrel 75 mg/day for 6months | Each patient had only one of the crural arteries treated. the implanted stents were 8–33 mm in length and 2.25–3.5 mm in diameter. |
| Tepe/2010/Germany | All patients received an 75mg clopidogrel and 100mg acetylsalicylic acid bolus prior to the intervention. During the procedure, 5,000 IU of heparin was given intra-arterially | acetylsalicylic acid 100 mg/day as continuous medication and clopidogrel 75 mg/day for eight weeks | none |
| Rastan/2011/Germany | Before the index procedure, all patients received oral aspirin (100 mg daily) and oral clopidogrel (a loading dose of 600 mg 24 h before the procedure) | aspirin 100 mg/day as continuous medication and clopidogrel 75 mg/day for 6 months | none |
| Bosiers/2012/multicenter European | Before the stenting procedure, patients received aspirin daily (75-300 mg) and clopidogrel (75 mg daily or by loading with 300 to 600 mg). | acetylsalicylic acid 75 mg/day and clopidogrel 75 mg/day or ticlopidine 250 mg/day for 12 months | none |
| Rastan/2012/Germany | Before the index procedure, all patients received oral aspirin (100 mg daily) and oral clopidogrel (a loading dose of 600 mg 24 h before the procedure) | aspirin 100 mg/day as continuous medication and clopidogrel 75 mg/day for 6 months | none |
| Scheinert/2012/in 9 European countries | Aspirin and a loading dose of 300 mg of clopidogrel (or 2 x 250 mg ticlopidine) were given preprocedure. Intraprocedural heparin was used to maintain activated clotting time levels >=250 s | low-dose aspirin and—in the SES arm only—75 mg clopidogrel (or 2x250 mg ticlopidine) were maintained for 6 months | patients in the SES group received up to 4 CYPHER SELECT stents (lengths 8 to 33 mm; diameters 2.5, 3.0, and 3.5 mm) within 1 vessel, or up to 2 stents per lesion in 2 different vessels, up to a total lesion length of 120 mm in a 1.1:1 stent-to-artery diameter ratio. |
| Siablis/2014/Greece | antiplatelet therapy prior to the intervention was not mentioned; During the procedure, 5,000 IU of heparin was given intra-arterially. | Patients were prescribed on dual antiplatelet therapy for 6 months. | DES used included zotarolimus-eluting (Resolute stent,Medtronic, Brescia, Italy; diameter: 3 to 4 mm, length: 30 mm), sirolimus-eluting (Cypher stent, Cordis, Bridgewater, New Jersey; diameter: 3 to 3.5 mm, length: 33 mm), and the everolimus-eluting stents (Promus stent, Boston Scientific, Natick, Massachusetts; diameter: 3 to 4 mm, length: #38 mm) according to department’s availability and were applied as a primary treatment (balloon pre-dilation as necessary). |
| Marlon/2016/ in 3 major vascular centers in the Netherlands | antiplatelet therapy prior to the intervention was not mentioned; During the procedure, 5,000 IU of heparin was given intra-arterially. | 100 mg of carbasalate calcium daily indefinitely and 75 mg of clopidogrel daily (with 300 mg loading dose) orally for ≥6 months | The full length of lesions was covered, if necessary, with overlapping stents. A maximum of 3 stents were allowed with a 3 to 5 mm overlap. |
| Marlon/2017/in 3 major vascular centers in the Netherlands | antiplatelet therapy prior to the intervention was not mentioned; During the procedure, 5,000 IU of heparin was given intra-arterially. | 100 mg of carbasalate calcium daily indefinitely and 75 mg of clopidogrel daily (with 300 mg loading dose) orally for ≥6 months | The full length of lesions was covered, if necessary with overlapping stents. A maximum of 3 stents were allowed with a 3 to 5 mm overlap. |
| Marlon/2020/in 3 major vascular centers in the Netherlands | antiplatelet therapy prior to the intervention was not mentioned; During the procedure, 5,000 IU of heparin was given intra-arterially. | 100 mg of carbasalate calcium daily indefinitely and 75 mg of clopidogrel daily (with 300 mg loading dose) orally for ≥6 months | The full length of lesions was covered, if necessary with overlapping stents. A maximum of 3 stents were allowed with a 3 to 5 mm overlap. |
| Siablis/2009/Greece | Patients were instructed to receive aspirin (100 mg/d) and clopidogrel (75 mg/d) for at least 3 days before the procedure. Otherwise, a loading dose of 300 mg of aspirin was administered the day of intervention. During the procedure, a bolus of 5,000 IU heparin was infused. | acetylsalicylic acid 100 mg/day and clopidogrel 75 mg/day for 6 months | Lesion treatment was then performed with appropriately sized angioplasty balloon catheters and stents in cases of suboptimal or complicated results of angioplasty. In cases of long or tandem lesions, more than one stent was placed in an overlapping manner (overlap of stent struts<5 mm). Inflow femoropopliteal lesions were treated as necessary. |
| He Tao/2015/China | 3 days before the procedure, patients received oral aspirin 100 mg/day and/or clopidogrel 75 mg/ day; During the procedure, 5,000 IU of heparin was given intra-arterially. | 5 days after the procedure, 5,000 IU of heparin was given every 12 hours.100mg aspirin and—in the SES arm only—75 mg clopidogrel were maintained for 6 months | none |

^[[1]](#footnote-1)^

1. SES: sirolimus-eluting stents; EES: everolimus-eluting stents; PES: paclitaxel-eluting stents; BMS: bare-metal stents; PTA: percutaneous transluminal angioplasty [↑](#footnote-ref-1)
