# Supplementary material for: Comparation of drug-eluting stents and control therapy for the treatment of infrapopliteal artery disease: a Bayesian analysis
Source: Int J Surg. 2023 Sep 14;109(12):4286–97. doi: 10.1097/JS9.0000000000000736 (PMC10720840; doi:10.1097/JS9.0000000000000736)
Supplement: SUPPLEMENTARY MATERIAL [file js9-109-4286-s011.docx]

The supplement digital content 7. The results of consistency test and inconsistency test for interest outcomes in the Bayesian analyses

| Outcome | Following period | Random Effects Standard Deviation | Inconsistency Standard Deviation |
| --- | --- | --- | --- |
| Clinical patency | 6 months | 0.73 (0.04, 1.64) | 0.84 (0.04, 1.65) |
|  | 1 year | 0.63 (0.06, 1.73) | 0.95 (0.05, 1.86) |
| Restenosis rate | 6 months | 0.91 (0.04, 3.04) | 1.64 (0.08, 3.22) |
|  | 1 year | 0.36 (0.02, 1.57) | 0.95 (0.04, 1.80) |
| Target lesion revascularizations | 6 months | 0.84 (0.01, 2.00) | 1.04 (0.05, 2.04) |
|  | 1 year | 0.70 (0.04, 1.76) | 0.92 (0.04, 1.80) |
| Ankle brachial index | 6 months | 0.05 (0.00, 0.10) | 0.05 (0.00, 0.10) |
|  | 1 year | 0.06 (0.00, 0.12) | 0.06 (0.00, 0.12) |
| All cause death | 1 year | 0.30 (0.02, 0.65) | 0.34 (0.02, 0.66) |
|  | 3 years | 0.37 (0.03, 0.96) | 0.51 (0.02, 0.98) |
| Amputation | 1 year | 0.33 (0.02, 0.71) | 0.36 (0.02, 0.71) |
