# Supplementary material for: Comparation of drug-eluting stents and control therapy for the treatment of infrapopliteal artery disease: a Bayesian analysis
Source: Int J Surg. 2023 Sep 14;109(12):4286–97. doi: 10.1097/JS9.0000000000000736 (PMC10720840; doi:10.1097/JS9.0000000000000736)
Supplement: SUPPLEMENTARY MATERIAL [file js9-109-4286-s012.docx]

**Identification of studies via databases and registers**

Records removed *before screening*:

Duplicate records removed (n =773)

Records identified from*:

PubMed (n =1620)

EMBASE (n=3842)

Cochrane (n=1158)

Registers (n =189)

**Identification**

Records screened

(n =6036)

Records excluded**

(n =6006)

Reports sought for retrieval

(n =30)

Reports not retrieved

(n =0)

**Screening**

Reports excluded:

BMS with special material (n=8)

Absorbable BMS (n=2)

Tack stent (n=1)

Lack of interest outcome event (n=2)

The patients’ lesions include other than infrapopliteal arterial disease (n=5)

Reports assessed for eligibility

(n =30)

Studies included in review

(n =12)

Reports of included studies

(n =12)

**Included**

*Consider, if feasible to do so, reporting the number of records identified from each database or register searched (rather than the total number across all databases/registers).

**If automation tools were used, indicate how many records were excluded by a human and how many were excluded by automation tools.

*From:*  Page MJ, McKenzie JE, Bossuyt PM, Boutron I, Hoffmann TC, Mulrow CD, et al. The PRISMA 2020 statement: an updated guideline for reporting systematic reviews. BMJ 2021;372:n71. doi: 10.1136/bmj.n71

For more information, visit: <http://www.prisma-statement.org/>
